# Supplementation of lupeol during in vitro maturation improves preimplantation embryonic development in pigs

**DOI:** 10.1016/j.vas.2026.100802

**Published:** 2026-08-09

**Authors:** Qingyi Lin, Takeshige Otoi, Oky Setyo Widodo, Theerawat Tharasanit, Kaywalee Chatdarong, Rentsenkhand Sambuu, Megumi Nagahara, Maki Hirata, Fuminori Tanihara, Zhao Namula

**Affiliations:** a Department of Veterinary Medicine, College of Coastal Agricultural Sciences, Guangdong Ocean University, 524088, Guangdong, China; b Faculty of Veterinary Medicine, Universitas Airlangga, 60115, Surabaya, Indonesia; c Bio-Innovation Research Center, Tokushima University, 779-3233, Tokushima, Japan; d Faculty of Veterinary Science, Chulalongkorn University, 10330, Bangkok, Thailand; e Institute for Extension of Advanced Agricultural Technology, 8098476, Ulaanbaatar, Mongolia

**Keywords:** GSH, Lupeol, Porcine embryo, ROS, Triterpene

## Abstract

Lupeol is a triterpene with several pharmacological properties, including antioxidant activity. This study investigated the antioxidant effects of lupeol supplementation during in vitro maturation (IVM) of porcine oocytes. Oocytes were divided into six groups and matured for 44 h in medium supplemented with lupeol at concentrations of 0, 1, 2, 4, and 8 µM. As a control, oocytes were cultured without lupeol or its dilution solution (dimethyl sulfoxide). After IVM, in vitro fertilization and embryo culture were performed to evaluate fertilization rate and developmental competence. Nuclear maturation, DNA integrity, intracellular glutathione (GSH) levels, reactive oxygen species (ROS) levels, fertilization, embryo development, and embryo quality were assessed. Lupeol at 4 and 8 µM increased metaphase II rates compared with the control group; however, the DNA integrity of the oocyte nucleus did not differ between groups. GSH levels increased, and ROS levels decreased at all concentrations compared with the control group. Monospermic fertilization rate of oocytes matured with 4 µM lupeol was higher than that of the 0 µM and control groups. Among the lupeol concentrations tested in IVM medium, only 4 µM significantly enhanced blastocyst formation. Moreover, 4 µM lupeol reduced DNA fragmentation in blastocysts compared with the 0 and 1 µM groups but did not affect the total cell number of blastocysts. In conclusion, the addition of 4 µM lupeol diluted in DMSO to the culture medium during IVM enhanced blastocyst formation by increasing intracellular GSH and reducing ROS in matured oocytes.

## 1 Introduction

Porcine in vitro production (IVP) systems, including in vitro maturation (IVM), in vitro fertilization (IVF), and in vitro culture (IVC), are widely used to produce large numbers of embryos for the generation of genetically modified pigs and other biotechnological applications. Despite various modifications to improve the efficiency of IVP, the developmental capacity of porcine *in vitro*-produced embryos remains lower than that of their in vivo counterparts (Dang-Nguyen et al., 2011; Kikuchi et al., 1999). Oocytes provide the molecular and cellular components to sustain embryo development until embryonic genome activation (Stitzel & Seydoux, 2007). Thus, embryo development highly depends on oocyte quality (Gosden, 2002; W.-H. Wang et al., 1999). Establishment of optimal IVM systems is required to produce high-quality in vitro embryos. In recent years, numerous researchers have attempted to improve IVM systems (Gil et al., 2010). However, the developmental capacity of IVM oocytes remains poorer than that of mature oocytes in vivo. Compared to in vivo conditions, in vitro culture systems present numerous barriers that can interfere with embryo development. The main drawbacks of these systems are the lack of antioxidants and a high concentration of reactive oxygen species (ROS) (Goto et al., 1993).

Among pentacyclic triterpenoids, lupeol is one of the most widely recognized members of this class, and it is a natural product widely consumed by humans through dietary resources such as fruits, vegetables, and medicinal plants (Ali et al., 2020; NA et al., 2017). Lupeol is a lipophilic pentacyclic triterpenoid that has attracted considerable attention because of its potent antioxidant, anti-inflammatory, and cytoprotective properties. Lupeol exhibits anti-inflammatory effects in *vitro* and in vivo (Geetha & Varalakshmi, 2001; Lambertini et al., 2005), suppresses the growth of various tumors in vitro and in vivo (Lee et al., 2007; Sunitha et al., 2001), and has demonstrated therapeutic potential in diabetes, cardiovascular diseases, and arthritis (Siddique & Saleem, 2011). In addition, lupeol has antioxidant properties, and supplementation of bovine IVM with lupeol improves embryo development and blastocyst quality (Khan et al., 2018). Previous studies have demonstrated that lupeol scavenges ROS, enhances endogenous antioxidant defense systems, and modulates oxidative stress-related signaling pathways, including the Nrf2/Keap1 pathway (Kim et al., 2024; Wang et al., 2024). Because oxidative stress is a major factor that impairs cytoplasmic maturation and developmental competence of oocytes during in vitro maturation, compounds with antioxidant activity are considered promising supplements to improve IVM systems. However, despite the well-documented antioxidant and cytoprotective properties of lupeol, its effects during porcine oocyte in vitro maturation have not been investigated. Moreover, the optimal concentration of lupeol for porcine IVM and its influence on oocyte maturation, intracellular redox status, fertilization, and subsequent embryonic development remain unknown.

Therefore, the present study aimed to evaluate the effects of lupeol supplementation during porcine IVM by assessing nuclear maturation, intracellular GSH and ROS levels, DNA integrity, fertilization, embryo development, and blastocyst quality. This study is the first to investigate the application of lupeol in porcine IVM and to identify an optimal supplementation concentration to improve embryonic developmental competence.

## 2 Materials and methods

### 2.1 General

Unless stated otherwise, all chemicals used in this study were purchased from Sigma-Aldrich (St. Louis, MO, USA).

### 2.2 Oocyte collection and IVM

Oocyte collection and IVM were performed as previously described (Lin et al., 2023). Briefly, ovaries of prepubertal crossbred gilts (Landrace × Large White × Duroc breeds) were collected from a local slaughterhouse and transported to the laboratory in physiological saline within 1 h at 30 °C. Cumulus-oocyte complexes (COCs) with uniformly dark-pigmented ooplasm and intact cumulus cell masses were collected from follicles using a surgical blade. Approximately 60 COCs were then cultured in 600 µL maturation medium, comprising tissue culture medium 199 with Earle's salts (TCM 199; Invitrogen Co., Carlsbad, CA, USA; Cat. No. 11150-059) supplemented with 10% (v/v) porcine follicular fluid, 0.6 mM cysteine (Sigma-Aldrich), 50 µg/mL gentamicin (Sigma-Aldrich; Cat. No. G3632), 50 µM sodium pyruvate (Sigma-Aldrich), 2 mg/mL D-sorbitol (Fujifilm Wako Pure Chemical Co., Osaka, Japan), 10 IU/mL equine chorionic gonadotropin (Celarmon 1000; Kyoritu Seiyaku, Tokyo, Japan), and 10 IU/mL human chorionic gonadotropin (Gestron; Kyoritu Seiyaku) for 22 h in a 4-well dish (Nunc A/S, Roskilde, Denmark) that was overlaid with mineral oil. The COCs were subsequently transferred into an IVM medium in the absence of hormones and cultured for an additional 22 h at 39 °C in a humidified incubator containing 5% CO_2_ in air.

IVM medium supplemented with different concentrations of lupeol (0, 1, 2, 4, or 8 µM), diluted in DMSO (Dojindo Laboratories, Kumamoto, Japan), was prepared prior to incubation. The COCs were divided into six groups and treated with lupeol at various concentrations to assess its antioxidant effects. As a control, COCs were cultured in IVM medium without lupeol or DMSO. The 0 µM group, containing only DMSO, was included to distinguish the effects of lupeol from those of the solvent. The DMSO concentration was 0.17% v/v for each experimental group.

### 2.3 Evaluation of oocyte nuclear maturation and DNA integrity

Nuclear maturation and DNA integrity were assessed in oocytes cultured in the presence or absence of lupeol using a combined technique for simultaneous nuclear staining with 4′,6-diamidino-2-phenylindole (DAPI) and terminal deoxynucleotidyl transferase nick-end labeling (TUNEL) adapted from the procedures previously described by Do et al. (2015).

Briefly, oocytes (20 per group) were denuded from cumulus cells using 150 IU hyaluronidase and mechanical pipetting. Denuded oocytes were fixed in 4% paraformaldehyde (Fujifilm Wako Pure Chemical Corp.) at 4 °C overnight and subsequently permeabilized with 0.1% (v/v) Triton-X100 for 40 min at room temperature. After permeabilization, fixed oocytes were incubated at 4 °C overnight in Dulbecco’s phosphate-buffered saline (D-PBS; Thermo Fisher Scientific, Waltham, MA, USA) supplemented with 10 mg/mL bovine serum albumin and followed by incubation in the dark with fluorescein-conjugated terminal deoxynucleotidyl transferase dUTP (TUNEL reagent; Roche Diagnostics Corp., Tokyo, Japan) for 1 h at 38 °C. TUNEL-stained oocytes were counterstained with 1 μg/mL DAPI (Thermo Fisher Scientific) and mounted onto glass slides. Labeled oocytes were examined using an epifluorescence microscope (Eclipse 80i, Nikon, Japan) fitted with epifluorescence illumination. Oocytes were classified as in the germinal vesicle (GV), condensed chromatin (CC), metaphase I (MI), anaphase I and telophase I (AT), or metaphase II (MII) stages based on chromatin configuration, as determined by DAPI staining. The proportion of oocytes with fragmented DNA was calculated by dividing the number of oocytes containing fragmented nuclei (labeled by TUNEL) by the total number of oocytes. Six replicates were used for each experiment.

### 2.4 Evaluation of GSH and ROS levels in oocytes

GSH and ROS levels were measured using two fluorescent probes: 4-chloromethyl-6,8-difluoro-7-hydroxycoumarin (CMF_2_HC, C12881; Thermo Fisher Scientific) for GSH levels and 2′,7′-dichlorodihydrofluorescein diacetate (H_2_DCFDA, D399; Thermo Fisher Scientific) for ROS levels. Briefly, approximately 30 denuded oocytes from each group were incubated in an IVM medium containing 10 µM CMF_2_HC or 10 µM H_2_DCFDA for 30 or 15 min, respectively. After incubation, the oocytes were washed with D-PBS containing 0.1% (w/v) polyvinyl alcohol (PVA) and placed in 10 µL microdrops. Images of fluorescently labeled oocytes were captured using a BZ-X710 fluorescence microscope (Keyence, Osaka, Japan) and saved as TIFF files. Two standard filter sets were used for the detection of ROS (excitation wavelength of 492–495 nm and emission wavelength of 517–527 nm) and GSH (excitation wavelength of 371 nm and emission wavelength of 464 nm). The fluorescence intensities of the oocytes were analyzed using ImageJ version 1.54 g (National Institutes of Health, Bethesda, MD, USA). Three replicates were used for each experiment.

### 2.5 IVF and evaluation of oocyte fertility

Frozen-thawed semen from the same batch collected from a single boar was used throughout this study. Within each experimental replicate, oocytes from all treatment groups were fertilized with the same thawed sperm sample to minimize variation in sperm quality among treatments. Before IVF, frozen-thawed spermatozoa were transferred into 5 mL of porcine fertilization medium (PFM; Research Institute for the Functional Peptides Co., Yamagata, Japan; Cat. No. IFP1020P), and washed by centrifugation at 500 × *g* for 5 min. After centrifugation, pelleted spermatozoa were resuspended in PFM and adjusted to a final concentration of 1 × 10^6^ cells/mL. Sixty matured oocytes were then transferred into each four-well dish (filled with 500 µL of PFM containing frozen-thawed spermatozoa per well and overlaid with mineral oil) and co-incubated for 5 h at 39 °C under 5% CO_2_, 5% O_2_, and 90% N_2_. After co-incubation, the presumptive zygotes were denuded and cultured in 500 µL of a porcine zygote medium (PZM-5; Research Institute for Functional Peptides Co.; Cat. No. IFP0410P), overlaid with mineral oil for 5 h at 39 °C in a humidified incubator containing 5% CO_2_, 5% O_2_, and 90% N_2_. PFM and PZM-5 were based on the formulations described by Yoshioka et al. (2008). Swim-up or density-gradient centrifugation procedures were not performed. Instead, spermatozoa were prepared by simple centrifugation and washing following our standard porcine IVF procedure. This approach was used because frozen-thawed boar spermatozoa prepared by washing provided stable fertilization outcomes in our IVF system. In contrast, additional sperm selection procedures may reduce sperm recovery and make it difficult to maintain a consistent insemination concentration in the extender.

Approximately 20 denuded zygotes were fixed and stained with acetic acid: ethanol (1:3, v/v) for 48–72 h. The fixed zygotes were stained with acetic orcein solution (1% orcein in 45% acetic acid) and examined under phase-contrast microscopy. Oocytes containing both female and male pronuclei were considered fertilized and categorized as monospermic or polyspermic according to the number of swollen sperm heads and pronuclei in the cytoplasm. Six replicates were used for each experiment.

### 2.6 IVC and evaluation of embryo development and quality

The remaining zygotes were cultured in PZM-5 medium for 3 days. All cleaved embryos were then transferred into 500 µL of a porcine blastocyst medium (PBM; Research Institute for the Functional Peptides Co.; Cat. No. IFP1030P), a modified PZM-5 optimized for blastocyst development, and cultured for an additional 4 days to evaluate their ability to develop to the blastocyst stage and the quality of the blastocysts. Six replicates were used for each experiment.

To evaluate the total cell number and DNA fragmentation in blastocysts, all embryos at the blastocyst stage were fixed at the end of the culture and analyzed using a combined technique of simultaneous nuclear staining with DAPI and TUNEL, as previously mentioned. The DNA fragmentation index was calculated by dividing the number of cells containing fragmented nuclei (labeled by TUNEL) by the total number of cells.

### 2.7 Statistical analysis

All statistical analyses were performed using STATVIEW (Abacus Concepts Inc., Berkeley, CA, USA). Data on oocyte nuclear maturation (GVBD and MII rates), DNA fragmentation, fertilization (total and monospermic fertilization rates), cleavage, blastocyst formation, intracellular GSH and ROS fluorescence intensities, blastocyst cell numbers, and the percentage of DNA-fragmented cells were analyzed using one-way analysis of variance (ANOVA), with lupeol concentration as the fixed factor. When ANOVA indicated a significant effect, differences among treatment groups were evaluated using Fisher's protected least significant difference (PLSD) post hoc test. Percentage data were arcsine-transformed before analysis. The normality of the data was assessed using the Jarque–Bera test before ANOVA. Data are presented as mean ± SEM, and differences were considered statistically significant at *P* < 0.05.

## 3 Results

### 3.1 Evaluation of oocyte nuclear maturation and DNA integrity

As shown in Table 1, supplementation with 4 µM and 8 µM lupeol significantly increased (*P* < 0.05) the proportions of oocytes that underwent germinal vesicle breakdown (GVBD) and reached MII compared with the control group; however, there were no differences in the DNA integrity of the oocyte nucleus between the groups.

**Table 1:** Effects of lupeol supplementation in the maturation medium on meiotic competence and DNA damage of porcine oocytes⁎.

Concentration of lupeol (µM)⁎⁎	Oocytes examined	No. (%) of oocytes with⁎⁎⁎		Oocytes examined	No. (%) of oocytes with DNA-fragmented nuclei
		GVBD	MII		
Control	113	104 (92.1 ± 2.8)^a^	60 (52.8 ± 8.4)^a^	130	2 (1.5 ± 1.0)
0	120	114 (95.0 ± 2.2)^ab^	69 (57.6 ± 5.0)^ab^	130	2 (1.5 ± 0.9)
1	117	112 (95.6 ± 2.1)^ab^	80 (68.2 ± 4.3)^ab^	128	0
2	118	112 (94.9 ± 1.4)^ab^	71 (59.8 ± 3.7)^ab^	133	1 (0.7 ± 0.7)
4	117	115 (98.3 ± 1.1)^b^	85 (72.8 ± 3.4)^b^	127	0
8	116	114 (98.3 ± 1.1)^b^	81 (70.2 ± 5.7)^b^	123	1 (0.8 ± 0.8)

a-bValues with different superscript letters in the same column are significantly different (*P <* 0.05).

⁎ Six replicate trials were performed. Data are expressed as the percentage of the mean ± SEM.

⁎⁎ As a control, oocytes were cultured in maturation medium without lupeol or its diluent (dimethyl sulfoxide).

⁎⁎⁎ GVBD, germinal vesicle breakdown; MII, metaphase II;.

### 3.2 Evaluation of GSH and ROS levels in oocytes

**Fig. 1:**
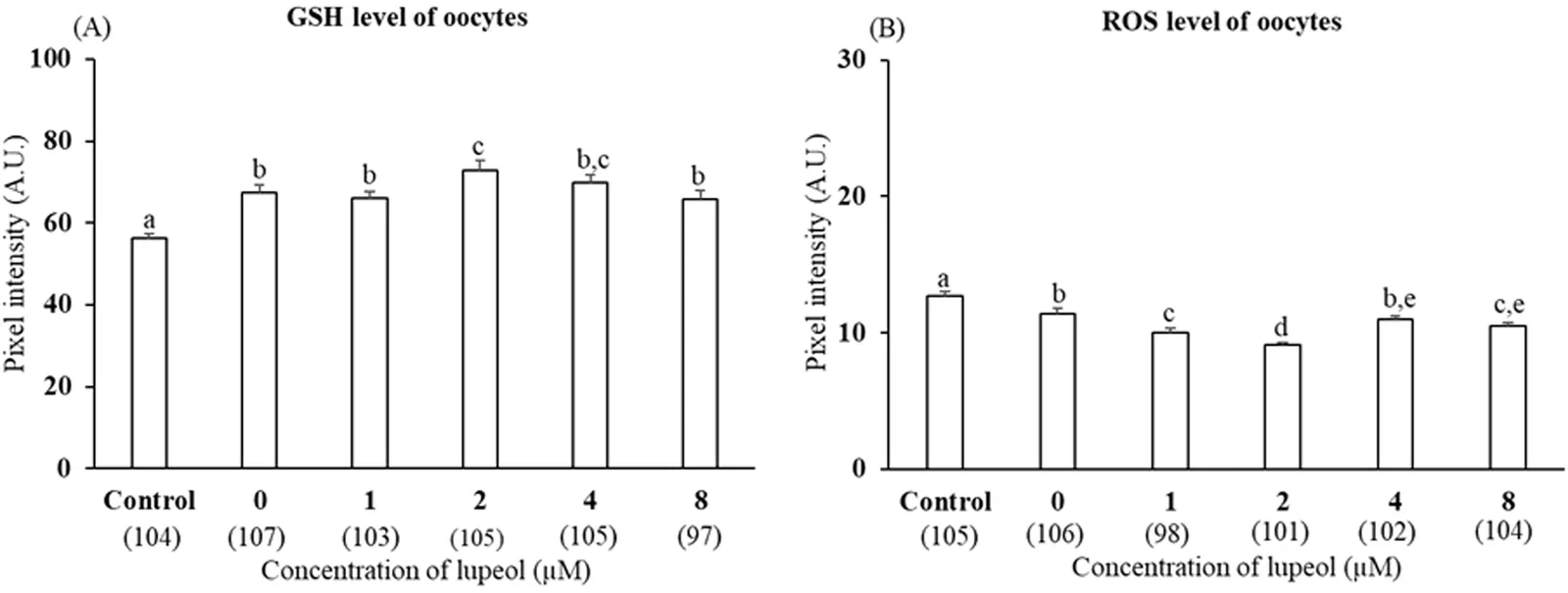
Effects of lupeol supplementation in the maturation medium on intracellular glutathione (GSH) and reactive oxygen species (ROS) in porcine oocytes. (A) GSH and (B) ROS levels in oocytes treated with various concentrations of lupeol during in vitro maturation, as measured by fluorescence intensity. As a control, oocytes were cultured in a maturation medium without lupeol and dilution solution (dimethyl sulfoxide). Numbers in parentheses on the x-axis indicate the total number of oocytes examined. Three replicate trials were conducted. ^a-e^Bars with different letters indicate significant differences (*P<* 0.05).

As shown in Fig. 1A, GSH levels significantly increased (*P* < 0.05) at all concentrations (0–8 µM), including the dilution solution group, compared to the control; however, only 2 µM lupeol significantly increased (*P* < 0.05) GSH levels in oocytes compared to the 0 µM group.

As shown in Fig. 1B, ROS levels significantly decreased (*P* < 0.05) at all concentrations (0–8 µM), including the dilution solution group, compared to the control group. Moreover, 1, 2, and 8 µM lupeol significantly decreased (*P* < 0.05) ROS levels compared to the 0 µM group.

### 3.3 Evaluation of oocyte fertility

**Fig. 2:**
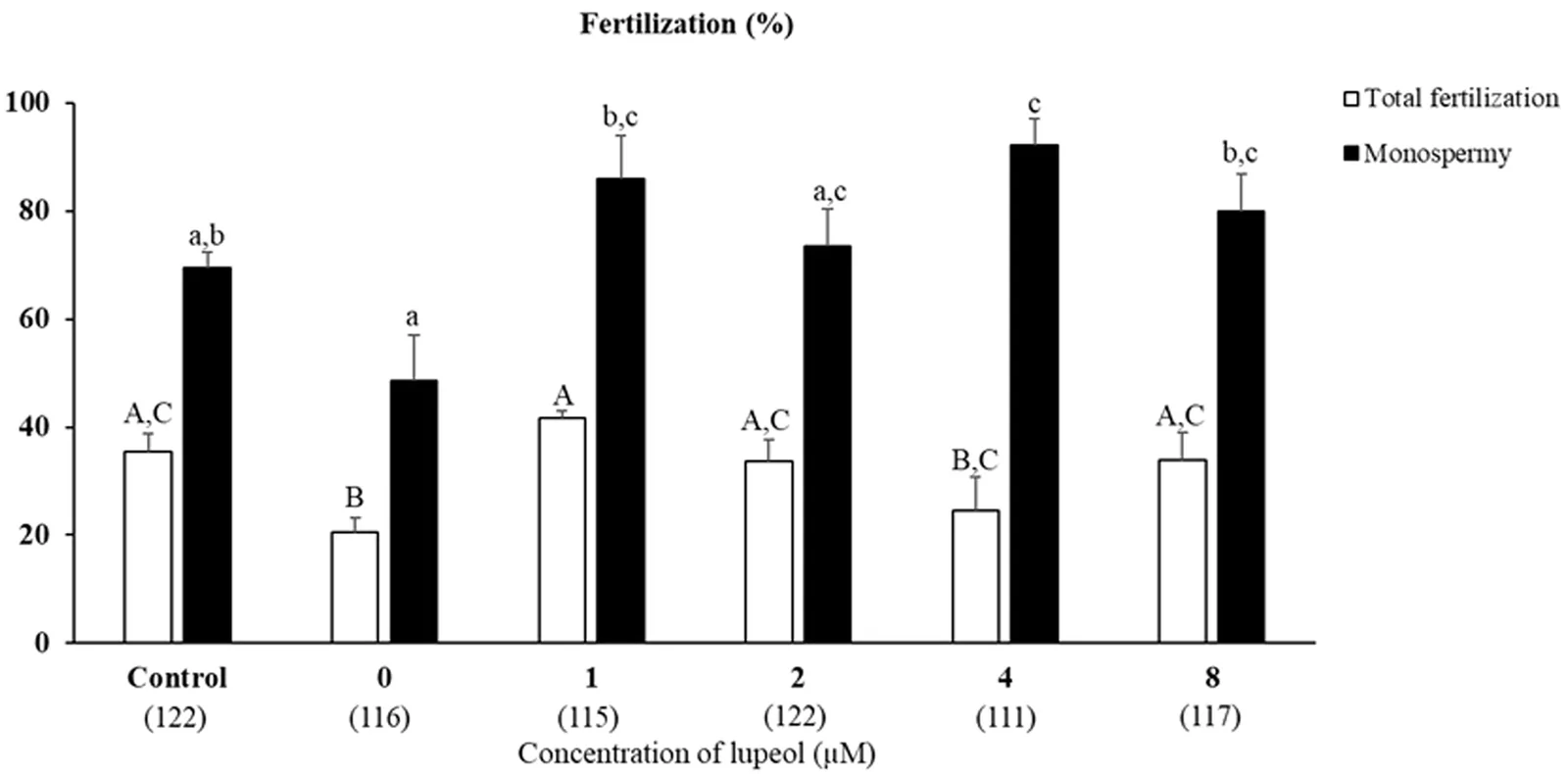
Effects of lupeol supplementation in the maturation medium on fertilization of porcine oocytes. As a control, oocytes were cultured in maturation medium without lupeol and dilution solution (dimethyl sulfoxide). Six replicate trials were performed. Data are expressed as the mean ± SEM. ^A–C^The total fertilization rate was defined as the ratio of fertilized oocytes to the total number of examined oocytes (the numbers in parentheses under the x-axis) with different superscripts that differed significantly (*P<* 0.05). ^a–c^ The monospermic fertilization rate was defined as the ratio of monospermic oocytes to the total number of fertilized oocytes; bars sharing different superscripts differ significantly (*P<* 0.05).

As shown in Fig. 2, the total fertilization rates of oocytes matured with 1, 2, and 8 µM lupeol during IVM were significantly higher (*P* < 0.05) than those of the 0 µM group. Moreover, the monospermic fertilization rate of oocytes matured with 4 µM lupeol was significantly higher (*P* < 0.05) than the 0 µM and control groups.

### 3.4 Evaluation of embryo development and quality

As shown in Table 2, supplementation with lupeol did not affect the percentage of cleaved embryos compared to the 0 µM group and the control, regardless of the concentration. Only 4 µM lupeol significantly improved (*P* < 0.05) blastocyst formation compared to the 0 µM and control groups. Moreover, the addition of 4 µM lupeol significantly reduced (*P* < 0.05) the proportion of DNA-fragmented cells in blastocysts compared with that in the 0 µM group. However, lupeol supplementation did not affect the total cell number of blastocysts.

**Table 2:** Effects of lupeol supplementation in the maturation medium on the development of porcine oocytes*.

Concentration of lupeol (µM)⁎⁎	Oocytes examined	No. (%) of embryos		Total cell number of blastocysts	% of DNA-fragmented cells
		Cleaved	Developed to blastocysts		
Control	242	210 (86.8 ± 1.8)^ab^	13 (5.4 ± 1.5)^a^	48.2 ± 3.6	4.2 ± 1.4^ab^
0	236	203 (86.1 ± 2.7)^ab^	20 (8.5 ± 1.5)^a^	46.3 ± 4.5	5.5 ± 1.4^b^
1	236	204 (86.3 ± 3.7)^ab^	20 (8.5 ± 1.5)^a^	40.5 ± 2.8	6.3 ± 1.4^b^
2	238	203 (85.2 ± 4.3)^ab^	22 (9.3 ± 2.6)^a^	43.1 ± 3.9	4.1 ± 1.2^ab^
4	227	209 (92.0 ± 1.6)^a^	34 (14.8 ± 1.6)^b^	42.8 ± 3.4	2.0 ± 0.5^a^
8	239	197 (82.6 ± 2.6)^b^	21 (8.8 ± 1.7)^a^	43.0 ± 3.9	3.4 ± 1.0^ab^

^a-b^Values with different superscript letters in the same column are significantly different (*P <* 0.05).

⁎ Six replicate trials were performed. Data are expressed as the percentage of the mean ± SEM.

⁎⁎ As a control, oocytes were cultured in maturation medium without lupeol and dilution solution (dimethyl sulfoxide).

## 4 Discussion

The ROS concentration is higher in vitro than in vivo (Goto et al., 1993). An imbalance between ROS and defense mechanisms against ROS affects cell maturation and subsequent development in vitro (De Lamirande et al., 1997). Supplementation with antioxidants is ideal for improving IVP in an environment that favors ROS formation. In this study, we found that 4 µM lupeol significantly increased the blastocyst formation rate, likely through enhancement of the MII rate and intracellular GSH accumulation, as well as reduction of ROS production compared to the control group. At this concentration, lupeol significantly increased the proportion of monospermic fertilization. The other tested concentrations (1, 2, and 8 µM) did not significantly improve blastocyst formation. Nevertheless, the addition of lupeol to IVM medium did not have deleterious effects, and almost all the results in blastocyst formation were superior to those of the control group.

In this study, we found that IVM medium supplemented with lupeol affected nuclear maturation. Supplementation with lupeol at 4 µM and 8 µM resulted in significantly higher GVBD and MII rates than the control group; however, there were no differences in oocyte nuclear DNA integrity among the groups. According to the study by Kim et al. (2024), triterpenes (e.g., lupeol) enhance meiotic maturation rates in porcine oocytes by regulating the Nrf2/Keap1 signaling pathway, which supports our findings of a higher MII rate. However, incomplete cytoplasmic maturation remains a serious problem associated with IVM (Sun et al., 2001), resulting in increased polyspermy and reduced developmental competence of IVM oocytes (W.-H. Wang et al., 1999). Intracellular GSH is a valuable indicator of cytoplasmic maturation (Luberda, 2005) and plays an important role in oocyte maturation. GSH also affects embryo development (Yoshida, 1993). In bovine IVM, antioxidant supplementation upregulates GSH synthesis and promotes development (Iwata et al., 1999). In porcine IVM, oleanolic acid (Wang et al., 2024) and betulinic acid (Kim et al., 2024), both triterpenoids, have been reported to increase intracellular GSH levels. In this study, all tested concentrations of lupeol in the IVM medium significantly improved intracellular GSH levels compared to the control group. Therefore, the addition of lupeol to the IVM medium may be sufficient to promote cytoplasmic maturation. Lupeol also significantly reduced ROS levels in mature oocytes compared with the control group. This reduction can be explained by the direct antioxidant role of lupeol and the concurrent increase in cytoplasmic GSH due to its supplementation in the IVM medium. It has been suggested that lupeol may also be important in oxidative defense systems (Khan et al., 2018). Moreover, the addition of lupeol to IVM medium significantly reduced the expression of the 8-OxoG gene, a ROS marker, in bovine oocytes (Khan et al., 2018). Although the mechanism by which lupeol reduces oxidative stress in porcine oocytes has not yet been defined, our results provide evidence that lupeol acts as an antioxidant, as GSH function in oocytes is primarily related to its antioxidant properties and protection against ROS (Gao et al., 2021; Kwak et al., 2012). Moreover, consistent with previous reports on antioxidant supplementation during porcine IVM, including resveratrol (Kwak et al., 2012), Indole-3-propionic acid (Nakayama et al., 2025), and melatonin (Kang et al., 2009; Shen et al., 2026; J. Wang et al., 2023), lupeol significantly increased intracellular GSH accumulation and suppressed ROS generation. The observed improvement in blastocyst formation rate and quality aligns with studies demonstrating that mitigating oxidative stress during maturation is critical for subsequent embryonic genome activation and cytoplasmic competence (Goto et al., 1993; Yoshida, 1993).

In this study, we found that the beneficial effect of lupeol on cytoplasmic maturation improves subsequent monospermic fertilization and embryo development. The addition of 4 µM lupeol to the IVM medium significantly increased the proportion of monospermic oocytes and the blastocyst formation rate compared with the control group. Although variations in GSH and ROS levels depended on lupeol concentration, the optimal concentration for embryo development was 4 µM. In bovines, 2 µM lupeol in the IVM medium significantly improved embryonic development (Khan et al., 2018). The optimal lupeol concentration differed from that reported in the present results, which may be due to species differences. Furthermore, GSH levels were comparable between the 2 µM and 4 µM lupeol groups, whereas ROS levels were lower in the 2 µM lupeol group than in the 4 µM lupeol group. An excessive reduction in ROS after lupeol treatment has been suggested to impair embryonic development, indicating that appropriate levels of ROS are required for embryonic development (Sakatani et al., 2007). Thus, 4 µM lupeol in IVM might be required to create appropriate in vitro conditions for porcine oocytes and embryos.

On the other hand, DMSO plays a dual role as either a pro-oxidant or an antioxidant, depending on its concentration (Pérez-Pastén et al., 2006; Sadowska-Bartosz et al., 2013). Low concentrations (0.1–0.5% v/v) of DMSO in bovine IVM medium did not harm embryo development, showing an increase in total GSH in blastocysts (Ynsaurralde-Rivolta et al., 2020). Studies have shown that low-dose DMSO has antioxidant effects by neutralizing the cytotoxic effects of ROS (Cederbaum et al., 1977; Shlafer et al., 1982). In this study, the DMSO group (0 µM) showed significantly increased intracellular GSH and decreased ROS levels compared with the control group, consistent with previous findings (Shlafer et al., 1982; Ynsaurralde-Rivolta et al., 2020). In addition, DMSO can reduce cellular inflammation by decreasing NF-κB activation (Han et al., 2023). Lupeol also reduces intracellular inflammation by downregulating NF-κB expression, thereby improving the development and quality of bovine embryos (Khan et al., 2018). Given that both DMSO and lupeol have been independently reported to suppress NF-κB activation, their combined presence in the IVM medium may have contributed to the observed improvement in blastocyst formation, though this warrants direct investigation.

## 5 Conclusions

Supplementation of IVM culture medium with 4 µM lupeol diluted in DMSO enhanced blastocyst formation. The addition of lupeol may increase the number and quality of transferable embryos by increasing intracellular GSH and reducing ROS during IVM. These findings collectively reinforce the potential of lupeol as a physiologically compatible IVM supplement. However, this study has several limitations. First, although lupeol supplementation improved intracellular redox status and embryonic development, the underlying molecular mechanisms were not investigated. Further studies are needed to elucidate the molecular mechanisms underlying the antioxidant effects of lupeol during porcine oocyte maturation, including the involvement of Nrf2/Keap1 and inflammatory signaling pathways. Second, the present study evaluated embryonic development only under in vitro conditions. Therefore, additional embryo transfer experiments are required to determine whether the beneficial effects of lupeol during IVM are maintained throughout pregnancy and result in the production of healthy offspring.

To our knowledge, this is the first study to demonstrate that lupeol supplementation during porcine IVM improves subsequent embryonic development by enhancing the intracellular antioxidant status. Unlike previous studies that focused mainly on bovine oocytes or other triterpenoids, the present study systematically evaluated multiple endpoints, including nuclear maturation, intracellular GSH and ROS levels, fertilization, blastocyst formation, and embryo quality, across a range of lupeol concentrations. These findings provide a scientific basis for incorporating lupeol as a novel antioxidant supplement in porcine IVM systems.

## Ethics approval

This study did not involve the use of live animals for experimental purposes. Porcine ovaries were obtained as by-products from a local slaughterhouse, where animals were slaughtered for commercial meat production and not specifically for this research. Therefore, no ethical approval for animal experimentation was required. All experimental procedures involving the collection and handling of biological materials were conducted in strict accordance with the ethical standards and guidelines approved by the Institutional Animal Care and Use Committee of Tokushima University, Japan (approval number: T2025-6).

## CRediT authorship contribution statement

**Qingyi Lin:** Writing – original draft, Investigation, Data curation. **Takeshige Otoi:** Writing – review & editing, Writing – original draft, Data curation. **Oky Setyo Widodo:** Writing – review & editing. **Theerawat Tharasanit:** Writing – review & editing. **Kaywalee Chatdarong:** Writing – review & editing. **Rentsenkhand Sambuu:** Writing – review & editing. **Megumi Nagahara:** Investigation, Formal analysis. **Maki Hirata:** Investigation, Formal analysis, Data curation. **Fuminori Tanihara:** Formal analysis, Conceptualization. **Zhao Namula:** Writing – review & editing, Conceptualization.

## Declaration of competing interest

The authors declare that they have no known competing financial interests or personal relationships that could have appeared to influence the work reported in this paper.

## Data Availability

The datasets supporting the conclusions of this article are included within the article.

## Appendix Supplementary materials

Image, application 1
