# Supplementary material for: Supplementation of lupeol during in vitro maturation improves preimplantation embryonic development in pigs
Source: Vet Anim Sci. 2026 Aug 9;33:100802. doi: 10.1016/j.vas.2026.100802 (PMC13521140; doi:10.1016/j.vas.2026.100802)
Supplement: Supplementary file 1 [file mmc1.pdf]

editage

Editing Certificate

Issued On May 28, 2026

This document certifies that the manuscript listed below has been edited to ensure language and grammar accuracy and is error free in these aspects. The edit was performed by professional editors at Editage, a brand of Cactus Communications. The author's core research ideas were not altered in any way during the editing process. The quality of the edit has been guaranteed, with the assumption that our suggested changes have been accepted and the text has not been further altered without the knowledge of our editors.

Manuscript Title

Supplementation of lupeol during in vitro maturation improves preimplantation embryonic development in pigs

Authors

Dr. Namula Zhao

GIUBW_54_2

Job Code

Prabh Grewal, Senior Vice President, Editage

QUALITY GUARANTEE • QUALITY GUARANTEE • QUALITY GUARANTEE

THE editage PROMISE

VERIFY AT
https://editage.jp/ec
verify

VERIFICATION CODE
EC-260528-
DMHQQZ
